# Beyond the null: Recognizing and reporting true negative findings

**DOI:** 10.1016/j.isci.2024.111676

**Published:** 2024-12-24

**Authors:** Manon K. Schweinfurth, Joachim G. Frommen

**Affiliations:** 1 School of Psychology and Neuroscience, University of St Andrews, St Andrews KY16 9JP, UK; 2 Department of Natural Sciences, Manchester Metropolitan University, Manchester M15GD, UK

**Keywords:** Health sciences, Medicine, Natural sciences, Biological sciences, Biological sciences research methodologies, Methodology in biological sciences, Interdisciplinary application studies

## Abstract

Science is based on ideas that might be true or false in describing reality. In order to discern between these two, scientists conduct studies that can reveal evidence for an idea, i.e., positive findings, or not, i.e., negative or null findings. The outcome of these studies can either be *true*, i.e., reflecting the real world, or *false*. Much has been said about disentangling true from false positive findings and the danger of a publication bias toward positive findings. Here, we argue that publishing negative findings is important to provide an accurate picture of the real world. At the same time, we highlight that a cautious approach should be taken to minimize the impact of publishing *false* negative findings, which has received limited attention so far. We discuss sources of false negative findings, using experimental and observational animal behavior and cognition studies as examples, which often differ from those of false positive findings. We conclude by recommending strategies for rigorous studies, such as conducting positive controls, selecting diverse samples, designing engaging protocols, and clearly labeling negative findings. These practices will lead to studies that contribute to our knowledge, regardless of whether they result in positive or negative findings.

## Graphical abstract

**Figure:**
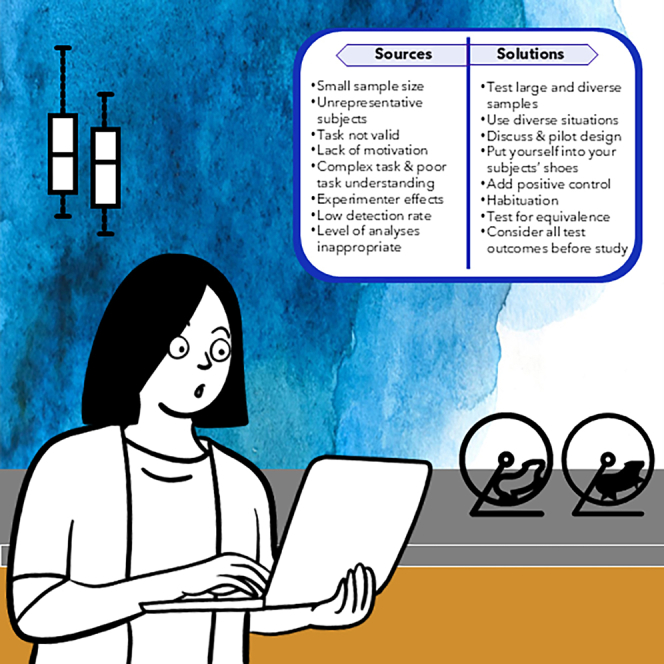

"Absence of evidence is not evidence of absence"

*Carl Sagan*^1^

Imagine a scenario that is too well known to many scientists. You had a fantastic idea, you designed the study, you got funding, you received ethical approval, you conducted the study, and the predicted effect is not there. Without doubts, this is a frustrating experience.

In the past, many such negative findings were not published, leading to a publication bias in the current literate, where publications report more positive findings than expected across disciplines.^2^^,^^3^ To tackle this underrepresentation in the literature, new journals were established that focused on publishing negative findings, e.g., the *Journal of Negative Results* or the *Journal of Negative Results in Biomedicine*. Today, some leading journals explicitly encourage publishing negative findings (e.g., *Nature Human Behavior* and *PLoS ONE* with examples such as^4^^,^^5^). As a result, the *Journal of Negative Results in Biomedicine* ceased publishing in September 2017 because they felt their mission was successfully completed.^6^

We highly welcome this development. Both positive and negative findings need to be reported to provide an accurate representation of the research field, rather than focusing merely on sensational or newsworthy findings. However, in order to be helpful in advancing the scientific field, negative findings need to be founded on a reliable body of evidence. Indeed, just like positive findings, negative findings might be true, when there is indeed no effect, or false, when there is an effect that was not detected (Figure 1A). Much has been said about the danger of publishing false positive findings^7^ and mitigation techniques have been implemented over the past years. Current open science practices, such as preregistering protocols and analyses prior to data collection as well as presenting peer review and raw data files openly, are aimed at detecting and reducing false positives in the literature. Yet, the pitfalls of publishing trustworthy negative findings have received less attention although they cannot be easily solved by following open science techniques. This oversight might be based on the perception that negative findings are more likely to be true because confirmatory biases or p-hacking can be excluded. Indeed, negative findings have become more prevalent in recent years, yet two out of three psychology articles reporting non-significant results contain evidence of at least one false negative^8^ and in >70% negative findings were misinterpreted.^9^ False negative findings can have dire consequences. For example, >30% of such erroneous claims in Educational Psychology could be linked to misguided educational theory, practice or policy.^10^

**Figure 1:**
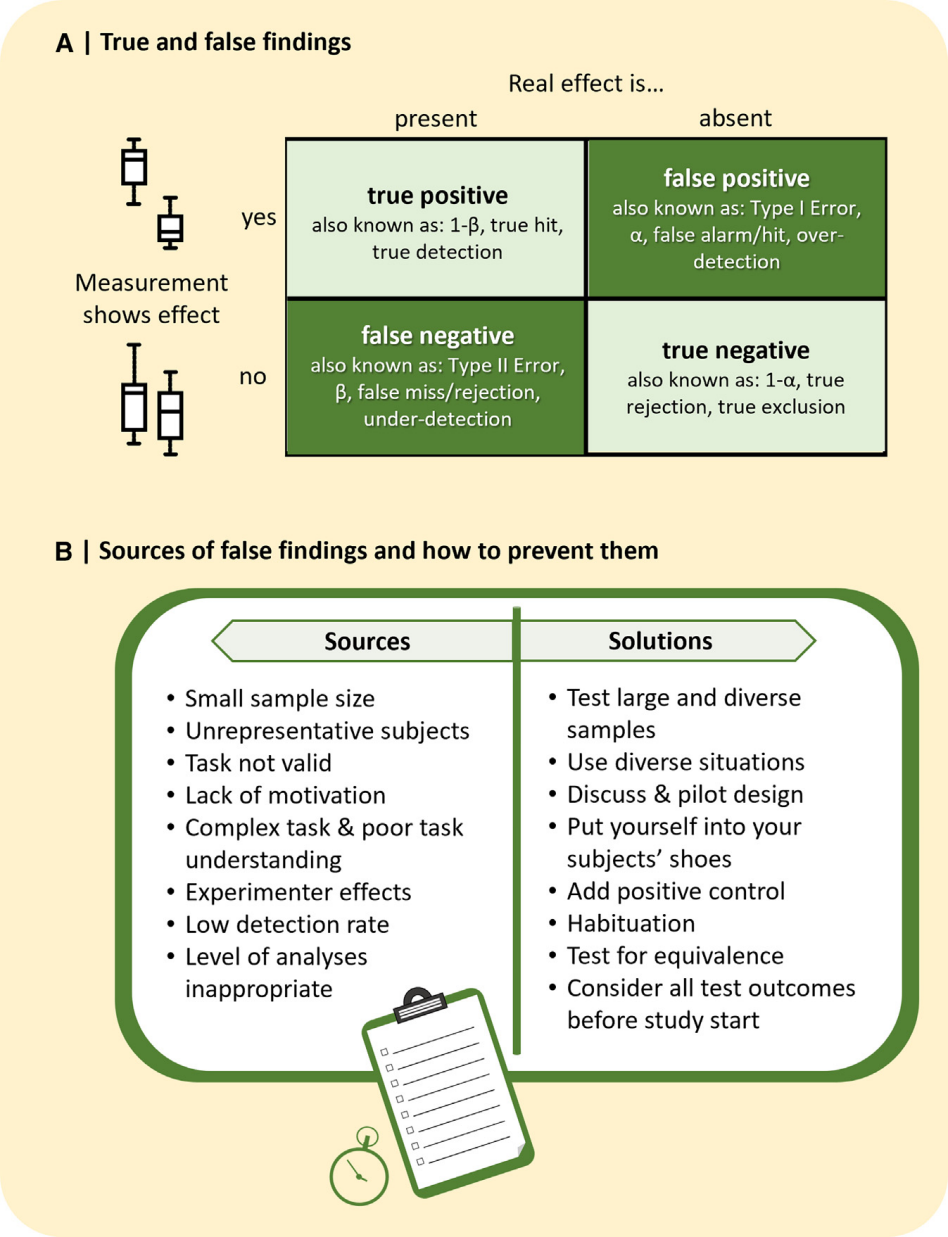
Navigating false negatives Studies result in true or false positive or negative findings, whereas synonymous terms are used in different fields (panel A). False negative findings can result from various sources, which can be prevented or mitigated (panel B).

Therefore, the aim of this article is to explore challenges and solutions for negative findings. We first highlight reasons for why negative findings might not be conclusive evidence for the absence of an effect, which might be different from distinguishing between true and false positive findings. We use examples from behavioral and cognitive research, which we will discuss for observational and experimental studies separately. While we focus on the fields where our own expertise is strongest, these issues can be generalized to other disciplines. Second, we will expand on why false negative findings are problematic and when negative findings should be reported. We conclude by making recommendations that will result in more conclusive findings, be they positive or negative.

## Reasons for false negative findings in experimental studies

Negative findings are not necessarily conclusive evidence for the absence of an effect because there can be various reasons why this seemingly negative (or null) finding might be false. Here, we provide a summary of the most common sources that we have encountered during journal clubs, peer reviews, and editorial work.

(1) **Sampled individuals might be too few.** The chance of detecting an effect depends on the effect and sample size. Studies that rely on only a few individuals will have low power to find an effect of low to medium size.^11^^,^^12^^,^^13^ Especially measurements that are characterized by high variation require large sample sizes to detect an effect. For example, a systematic literature review revealed that only 10–20% of studies conducted in the field of Animal Behavior exceeded a statistical power of 80%, i.e., the probability of obtaining a significant result when the effect is true.^14^ Additionally, studies are often biased toward a small pool of species,^15^ limiting generalizability of effects across phylogeny. It is hence unclear if a study reports no effect because there is no effect, which would be a true negative finding, or whether it lacks the power to detect an effect, which would be a false negative finding.^13^^,^^16^.
(2) **Sampled individuals might not be representative.** Some research fields are biased toward certain individuals especially when working with long-lived species, as these individuals are often probed repeatedly in different studies, leading to non-independent samples.^15^ It is important that test subjects – especially when working with few individuals of a given species repeatedly - are representative for their species or at least population, which is not trivial to assess.^17^^,^^18^ For instance a multi-site comparison of primate working memory abilities showed that individuals of the same species that were living at different sites performed differently, which might be the result of different levels of experiences with such tasks.^19^ In addition, it was found, for instance, that a lack of predation and parasites in the lab boosts the immune response of guppies (*Poecilia reticulata*), which changes their behavior.^20^ Hence negative findings on a sample might not be generalizable for the entire species.
(3) **The study protocol may not adequately assess the concept it claims to test.** Scientific studies that make use of non-human subjects are necessarily planned from a human perspective. However, study animals might perceive the world in a fundamentally different way. Hence, while the experimenter designs a protocol that can be validated by human subjects, the set-up might not be meaningful for the test animals. For example, many fish species see light in the ultraviolet spectrum^21^ and use ultraviolet coloration to communicate during mate choice^22^^,^^23^ and social interactions.^24^^,^^25^ As human researchers lack this ability, we tend to design studies excluding UV light. Finding no evidence for the use of visual cues in certain interactions of fishes might therefore be because not all necessary visual cues were available to them.
  In addition to perceptual differences, species differ in their likelihood to perform behaviors under some social settings. For example, many studies have aimed to test whether one of our closest living relative, the chimpanzee (*Pan troglodytes*), shows evidence for Theory of Mind, which is the ability to ascribe mental states to others like “I know what you know.”^26^ After years of research and using different protocols, it had been concluded that chimpanzees do not have a Theory of Mind.^27^ This false negative finding was revealed by an innovative study, which used a competitive rather than a cooperative setting, demonstrating that chimpanzees after all are able to know what others know.^28^ Probably, the discovery was hindered as humans frequently apply the Theory of Mind in cooperative settings.^29^ Hence, older studies did not test the *general occurrence* of Theory of Mind, as intended by the researchers, but instead Theory of Mind *under very specific conditions*. Nowadays, it is well established that chimpanzee have a Theory of Mind if tested in the right setting.^30^.
(4) **The study design might not be motivating enough for individuals to respond.** Many studies provide a form of stimulus to elicit an expected result in the test subjects. Such stimuli might for example include the presence of conspecifics^31^ or food.^32^ Yet, designing a task that is motivating for a range of test subjects with different backgrounds is not trivial. For example, while food is used regularly as a reward for successful trials, the value of food types differs for individuals based on hunger levels,^32^^,^^33^ individual preferences^34^ cultural differences,^35^ or previous experience.^36^^,^^37^ Hence, not all test subjects might be equally motivated to partake, leading to noise in the data. If the motivation is too low, subjects might not respond at all or only some individuals will respond. Additionally, if the motivation to partake is too high, attention to details and impulsivity control might be reduced,^36^ also leading to false negative findings.
(5) **The study design might be too complex to understand.** For certain questions, for instance to rule out previous experience, it can be important to expose subjects to novel (and hence often artificial) tasks, settings, or objects. However, this can be unintuitive for participants, especially when participants are not human.^38^ For instance, chimpanzees are more likely to be prosocial toward their conspecifics in less complex and more naturalistic experimental tasks.^39^ Similarly, the performance in a spatial navigation task of three-spined stickleback (*Gasterosteus aculeatus*) and minnow (*Phoxinus phoxinus*) has been shown to be dependent on task complexity,^40^ exemplifying the risk that overly complicated mazes lead to the erroneous assumption of the absence of certain cognitive abilities in these species. Hence, to conclude that an effect is absent requires experimental proof that the test animal understood the task. Yet, not all studies reporting negative findings demonstrate successful task-understanding controls (reviewed in^41^^,^^42^).
(6) **Experimenters or environments might prevent individuals from responding according to their natural or best performance by disturbing or distracting them.** Lab studies often include interactions with the test subjects, which can include catching, carrying, or placing them into a testing arena. All of these interactions can induce stress and affect their behavior,^43^ bearing the risk of confusing a true negative finding with a test outcome caused by neophobia or fear. To avoid anxiety, researchers use habituation techniques, in which test subjects are exposed to test conditions prior to the test. For example, spotted rainbowfish (*Melanotaenia duboulayi*) that were habituated to the study protocol and testing arena show better escape responses toward novel trawl apparatuses compared to when they were not habituated.^44^ However, habituation is not a straightforward solution and contains a strong species- and setup-dependent component.^45^ For example, agonistic behaviors of social cichlid fish shown toward a mirror decrease when they got habituated to the experimental setting,^46^ probably because mirror images cannot harm test subjects in contrast to real opponents.^47^ Consequently, several species, including fishes, eventually learn to use their mirror image to inspect their own body instead of fighting it.^48^^,^^49^ In such cases exposing the test individual to the experimental setting for too long might be the cause for false negative findings if one is interested in aggression. Yet, some skills can only be revealed after extensive training and habituating phases, such as chimpanzees showing evidence to understand human words after years of language training^50^
  Sub-optimal acclimation is not the only source of creating false negative results. Other habituation effects impact study results, too. Carrion crows (*Corvus corone*) and ravens (*Corvus corax*), for example, are more likely to participate and succeed in a cognitive task with a familiar compared to an unfamiliar experimenter,^51^ highlighting the role of a relationship between experimenters and test subjects. In contrast, switching experimenters during a study on Montagu’s harrier (*Circus pygargus*) chicks decreased stress levels and aggressive behaviors.^52^ In addition to experimenters, social environments can impact subjects. For example, highly social orange-winged amazons (*Amazona amazonica*) are more likely to participate in a behavioral task when tested in a social compared to an individual setting.^53^ All these inter- and intraspecies social effects can make it more difficult for test subjects to perform well and hence might lead to false negative findings.

## Reasons for false negative findings in observational studies

Observational studies differ from experimental studies in that they usually interfere less with the study subjects and often happen under less controlled conditions. As a result, false negative findings can be caused by factors that differ from lab studies, which we will discuss below. Publication bias toward positive findings is difficult to assess but might be more pronounced in observational compared to experimental studies because observational studies are often based on multiple observations instead of a few pre-defined responses to a certain task in experiments. As a result, it is unlikely that one picks a negative over a positive finding as the basis for a publication. Furthermore, findings from observational studies can rarely be fully replicated due to social and ecological changes in the test population or population differences^54^ and hence the frequency of false positive and negative findings are hard to estimate. Nonetheless, the absence of a trait can be an important finding, and we encourage reporting them, as long as it is unlikely that the finding might be false.

(1) **The sample size might be insufficient.** Although observational studies are often based on larger sample sizes than experimental studies, some patterns can only be revealed when studying a large number of individuals of a species. For example, only when the behavior of chimpanzee groups all over Africa was compared, traits were revealed that suggested that non-human animals have culture.^55^ Hence, comparable to under-powered lab studies, conclusions about the absence of traits from non-representative samples should be drawn with caution.
(2) **The sample might not be representative.** While it is often feasible to directly observe all group members in captive settings, this is more difficult in the wild. Personality and rank differences can affect habituation and hence observability of certain group members.^56^ Furthermore, it is important to ensure that not only conspicuous groups or traits are studied, as this can bias the literature.^57^ Likewise, populations differ, and what is found in one population might not be generalizable to the entire species. Carrion crows, for example, are described as pair-breeding throughout most of their range. Still, there are populations in which breeders regularly accept brood-care helpers at their nest, challenging pair-living as the only social structure in this species.^58^ Unrepresentative samples might therefore lead to false negative findings that cannot be generalized to the entire species.
(3) **The detection rate to demonstrate an effect might be too low.** In order to exclude false negative findings, subjects need to be studied under diverse circumstances, including different temporal (like the entire time of the day and season) and spatial resolutions (like the entire ecological niche of the individual and species). For example, honey-dipping tools were only discovered in West African chimpanzees after using direct (camera traps) and indirect (collection of abandoned tools) observational techniques over 23 consecutive months in four different communities.^59^ Further, the observation length can impact detection rates. For example, decades of data collection over multiple generations can be necessary to relate life-history data to behavioral traits and cognitive skills.^60^^,^^61^ Similarly, to analyze patterns in rare behaviors, long-term datasets are needed. For example, 40 years of research were needed to document rare cases of chimpanzee mothers carrying their long deceased infant, allowing for suggestions about the mechanisms and functions.^62^ Finally, observer presence can disturb the natural behavior of individuals and hence reduce detection rates of certain skills or behaviors, as shown in Colombian white-faced capuchin (*Cebus capucinus*).^63^
(4) **False negative findings might be a result of masking effects and missing variables.** Studying animals in their natural environment has the benefit of measuring behaviors that are meaningful to the subjects in their natural context. However, a drawback is that it is difficult to focus on single effects, as there is a virtually endless number of potential co-effects and confounding factors. Some effects can be easier to measure than others and hence may mask relationships with the latter that are harder to detect. For example, a simulation study on cooperation networks demonstrated that provided help can be explained by kinship and reciprocity, but because nepotism can be more easily and reliably detected, it often masks the effect of reciprocity on helping decisions, especially when the sample size is small.^64^ Hence studies with a small sample size might conclude that an effect is of minor importance or absent while it is merely masked by a co-effect and hence a false negative finding.
  In addition to masking effects, missing variables can lead to false negative findings. Relationships are almost always a result of several factors and hence they might not be easily detectable without knowledge of more than a few parameters. For example, in several territorial vertebrate species territoriality can only be meaningfully observed if several parameters are measured at the same time, e.g., intruder displacement in addition to site fidelity.^65^

## Reporting negative findings

There is little doubt that the scientific endeavor strongly benefits from the publication of true negative findings. Not publishing true negative findings can impact meta-analyses by overestimating effect sizes,^66^ and can lead to the establishment of false positive facts.^67^ This can be illustrated by the following example: If a study has been conducted 20 times and only one repeat resulted in a positive finding, it is most likely a statistical artifact. However, this artifact could not be detected as such without the publication of at least some of the other 19 studies, which put the one positive finding into context.^68^

Given this risk, one could argue that it is always important to publish negative findings, regardless of whether true or false, because they may contain some information. This implies that there is always something to learn from negative findings, even if it is just an instruction of “how to not do it.”^69^ The problem is that there are often multiple reasons why something did not work out. As a result, it is almost impossible to assess which part of a study “not to do” in the future, especially if the study was not carefully executed in the first place. For example, a study that did not result in any behavioral change can have multiple reasons. The setup might not be valid, or it is valid, but the test subjects were unrepresentative, the training was inappropriate, task understanding was poor, and so forth. On top and more difficult to detect, many studies need considerable knowledge about the model species, handling techniques, or the experimental set-up which researchers only gain with experience. In such scenarios, learning how to conduct a study or observe certain traits might require extensive training and can affect study results.^70^^,^^71^

We think a far better approach than publishing every finding is to empower researchers to distinguish between true and false negative findings and publish only true findings in academic journals for several reasons (Figure 1B). First, false negative findings can have detrimental effects - sometimes even more than false positive findings. For example, a meta-analysis suggested that studies in Animal Welfare that aimed to assess the adversity of events, such as transporting live animals, are often underpowered and hence have a higher chance of resulting in false negative findings with potential severe wellbeing implications.^72^ Second, if every study, whether true or false, is published, it will be difficult to discriminate between these findings, known as the “cluttered office” effect.^73^ In 2016, it was estimated that two articles per minute were published - in the biomedical sector alone.^74^ The more studies are available, the more difficult it is to keep track of every publication and the less time each researcher has available to assess their validity, which is especially problematic when the article covers a species or discipline that the reader is less familiar with. Third, publishing every dataset means that more false (positive and negative) findings are published, which impacts the overall quality of science.^75^ This does not necessarily mean that findings that bear the risk of being false could not be published at all. They might be submitted to specialized journals^69^ or online platforms to avoid any confusion with true findings. However, it should be emphasized that no firm scientific conclusions should be drawn from such studies.

Therefore, we advocate that only those negative findings should be reported in peer-reviewed journals that provide evidence that they are likely to be true, for which we give concrete recommendations below. The file-drawer effect, which describes a systematic bias in scientific research to not publish negative findings, is harmful to science. However, there are good reasons to file studies that bear the risk of being false, and it needs expert knowledge to discriminate between such studies rather than indiscriminately publish positive and file negative findings. Better than filing any studies, however, is to design rigorous studies that control for common reasons why negative findings might be false. This is not only beneficial in terms of costs and time, but also avoids exposing subjects unnecessarily to experimental manipulations or repeated observations.

## Recommendations

When designing a study, scientists tend to think about alternative explanations for positive findings and conduct control experiments to rule these out. While this is good practice, we encourage scientists to also think about alternative explanations for a negative finding and how one could be confident that a negative outcome reflects true effects. Many best-practice recommendations emphasize the danger of false positive findings with a strong focus on minimizing them. Open science practices are most effective in reducing the likelihood of false positives by preventing p-hacking or data dredging, for instance. However, while reducing the number of false positive findings is crucial, false negative findings are also an issue that must not be overlooked. While open science practices primarily focus on mitigating false positive findings, openly deposited methods, analyses, and data can also reveal positive effects that were missed, thereby contributing to identifying false negatives. Nevertheless, open science practices alone cannot fully address the risk of false negative findings. In the following, we list some suggestions that are relevant before, during, and after conducting a study and go beyond open science practices. Note that this list is not exhaustive and is aimed at stimulating further thoughts dependent on the research question, study species, and testing environment.

**Conduct positive controls to ensure the study tests what it claims.** If the study revealed a negative result, it is important to demonstrate that the treatment itself worked. It might be that the treatment is insignificant for test subjects, they are distracted, or not motivated to respond. For this, it is important to include a positive control, which ensures the experimental setup is functioning as intended. Such a control can rule out many possible confounding effects and may take various forms. It can replicate earlier findings or show an expected response by the test subjects toward the stimuli. Specifically, it shows that the experimenter and the setting can produce meaningful data and that subjects perceive the stimuli and are motivated to respond. For example, a study on magpies (*Pica pica*) investigated whether the birds are attracted to shiny objects.^76^ Birds were tested in captivity and in the wild, but none of the birds was attracted to shiny objects. A positive control condition revealed, however, that the birds paid attention to provided items and readily picked up food that was next to the objects. Hence, the researchers could demonstrate that the set-up itself worked, but that contrary to common beliefs magpies appeared to show no significant attraction to shiny objects. Furthermore, and especially in rather artificial tasks, it is crucial to test for full task understanding by setting up probing trials and including only those subjects in the test that passed them. For example, a study with an artificial food-provisioning task revealed reciprocal cooperation in chimpanzees only in those who passed the task understanding controls.^77^ Finally, we think it is important to share and discuss the study protocol with peers, which can be done in seminars, at conferences, or via pre-registrations. Receiving feedback and discussing alternative explanations is much more productive before than after conducting a study, which is the case for peer-review because the protocol can be easily refined at an earlier stage. Here, discussions should range from all possible study outcomes, positive or negative, to seemingly minor protocol details, like randomizations and participant selection, to cover ideally all aspects of the study. The ARRIVE guidelines provide a good starting point and their application has been suggested to improve the quality of studies.^78^

**Aim for appropriate sample sizes in order to provide stronger evidence for a negative finding.** It is part of science that ideas turn out wrong and no effect of a treatment can be found. In this case, it is important to assess whether the lack of a treatment effect is based on a study that lacks the power to detect a true effect - false negative finding - or whether test subjects show indeed a similar response to the treatments - true negative finding. Depending on whether one follows a Frequentist or Bayesian approach, the options differ.^79^ Following the Frequentist approach, equivalence tests should be conducted to assess whether responses are indeed equal,^14^ confidence intervals should be used to provide parameter estimates with degrees of certainty,^80^ and power should be calculated to assess whether the sample size is too small to detect a difference.^81^^,^^82^^,^^83^ Alternatively, the Bayesian approach enables a more nuanced understanding of the data, including a level of confidence, using Bayes Factors,^84^ and more direct support of no differences between two treatments,^85^^,^^86^ especially when dealing with small sample sizes.^87^

**Use representative samples and report their background.** During data collection, it is important to generate meaningful and valid data for which a diverse pool of subjects is needed that show their undisturbed and representative behavior. Individuals differ and the more a researcher reduces or “standardises” the subject pool and their environment, the less generalizable are the study outcomes, which can lead to poor reproducibility.^17^^,^^88^^,^^89^ It is therefore crucial to keep the source of variability in mind (see^18^ for detailed discussion). For example, it can make a difference whether individuals are related (or even inbred), had early life experiences that make them aversive to a task, have already been tested and therefore have been influenced by other experiments, or are bonded to the human observer (see above). Also, different rearing and keeping conditions have to be kept in mind when concluding that a result is a true negative.^90^^,^^91^ While some variation can be achieved by simply keeping animal subjects under more diverse conditions,^92^ other factors, such as social background and rearing history, are admittedly more difficult to diversify.^18^ This can be achieved by collaborations between labs.^93^^,^^94^ Likewise, before concluding that a certain trait or behavior is absent, it is crucial to ensure that this absence is not just based on population differences or can be explained by limited chances to observe a behavior in a population.

**Make sure the study protocol is engaging for individuals.** Before conducting any study, it is important to design a set-up that is motivating and relevant to the test subjects. To do so, it might be helpful to put oneself into the shoes of a subject and consider what they perceive and encounter in their given environment, how they would react naturally, and whether the stimuli would matter to them in their normal life.^95^ For example, despite many years of research, there was no convincing evidence that non-human primates would understand false beliefs. Only after exposing them to a human in an ape costume, this skill could be revealed.^96^ In addition, by testing animals in their natural environment or by mimicking real-world circumstances, ecological validity and hence relevance can be increased.^97^

**Consider whether the individuals had a fair chance to show the expected results.** Using complimentary testing protocols can provide insights into whether subjects are unable to solve a task generally or just in a certain setting.^98^ In addition, caution should be paid to habituating individuals to testing environments or the presence of an observer. Just like learning criteria, experimenters should *a priori* define thresholds that suggest appropriate habituation while avoiding overstimulation to gather valid data (see above). Further, one has to ensure that detection rates are high to observe the trait of interest. This can be achieved by testing individuals under different spatial and temporal resolutions,^99^ using extended periods of observer presence^100^ or using novel technology to avoid the presence of observers.^101^ Technical advancements have created exciting and less time-consuming opportunities to observe individuals over time and spatial scales, which have so far been impossible to explore via direct observations. This has resulted in astonishing studies from tiny hoverflies migrating at high altitudes^102^ to giant Humboldt squids (*Dosidicus gigas*) living in the deep sea.^103^ Still, it is important to note that not all data can be remotely collected for extended periods for which direct observations can be indispensable.^104^

**Clearly label negative findings.** A study that analyzed >200 articles in the field of Animal Cognition found large heterogeneity in how non-significant effects were labeled, including various instances of i) ambiguous and imprecise wording and ii) misinterpretation of non-significant results as support for the null hypothesis, the latter being prevalent in >80% in titles.^105^ Similar effects were also found in other fields.^9^^,^^106^ Therefore, if a non-significant result is obtained in a study, the finding should be clearly labeled, such as “The analysis did not show a significant effect of the manipulation” and effect sizes and/or confidence intervals should be discussed to increase transparency. Statements such as “A was similar to B” or “There was no effect of A on B” are not justified based on non-significant results because a failure to reject H_0_ does not confirm its correctness or generalizability. When reporting negative findings, one should not exclude the possibility of large individual variation, meaning that other individuals in a different setting might have provided a positive finding. Probably the most extreme example is the gray parrot (*Psittacus erithacus*) Alex. Although just one individual, much has been learned from him about numerical abilities and abstract abilities, thereby showing the potential capabilities of a species.^107^

## Conclusions

Negative findings are valuable findings and fundamental to better understanding the world around us - they are part of all scientific discoveries. However, negative findings can be true or false, and one needs to be cautious to not confuse them, as false negative findings can lead to wrong conclusions. Here, we highlighted causes that can lead to false negative findings with the aim to start a discussion on how to distinguish true from false negative findings and when (and how) to report them. We hope that our recommendations will be helpful for designing rigorous studies that result in conclusive findings, independent of whether they are positive or negative.

## Acknowledgments

MKS acknowledges funding from the 10.13039/501100000268Biotechnology and Biological Sciences Research Council (grant number: BB/X00631X/1). We would like to thank three anonymous reviewers for their thoughtful comments on our article.

## Author contributions

MKS and JGF conceived the work; MKS wrote the first draft of the article which was expanded by JGF. MKS and JGF approved the final version of the article, which MKS submitted.

## Declaration of interests

The authors declare no competing interests.
